# Sex-Specific Effects of Microglia-Like Cell Engraftment during Experimental Autoimmune Encephalomyelitis

**DOI:** 10.3390/ijms21186824

**Published:** 2020-09-17

**Authors:** Jinming Han, Keying Zhu, Kai Zhou, Ramil Hakim, Sreenivasa Raghavan Sankavaram, Klas Blomgren, Harald Lund, Xing-Mei Zhang, Robert A. Harris

**Affiliations:** 1 Applied Immunology and Immunotherapy, Department of Clinical Neuroscience, Karolinska Institutet, Center for Molecular Medicine, Karolinska University Hospital, S-171 76 Stockholm, Sweden; keying.zhu@ki.se (K.Z.); harald.lund@ki.se (H.L.); xingmei.zhang@ki.se (X.-M.Z.); 2 Department of Women’s and Children’s Health, Karolinska Institute, Karolinska University Hospital, S-171 76 Stockholm, Sweden; kai.zhou@ki.se (K.Z.); klas.blomgren@ki.se (K.B.); 3 Department of Clinical Neuroscience, Karolinska Institutet, Karolinska University Hospital, S-171 76 Stockholm, Sweden; ramil.hakim@ki.se (R.H.); Sreenivasa.raghavan@gmail.com (S.R.S.); 4 Pediatric Oncology, Karolinska University Hospital, S-171 76 Stockholm, Sweden; 5 Department of Physiology and Pharmacology, Karolinska Institutet, Center for Molecular Medicine, Karolinska University Hospital, S-171 76 Stockholm, Sweden

**Keywords:** microglia, microglial repopulation, monocytes, neuroinflammation, experimental autoimmune encephalomyelitis

## Abstract

Multiple sclerosis (MS) is a chronic neuroinflammatory disorder of the central nervous system (CNS) that usually presents in young adults and predominantly in females. Microglia, a major resident immune cell in the CNS, are critical players in both CNS homeostasis and disease. We have previously demonstrated that microglia can be efficiently depleted by the administration of tamoxifen in *Cx3cr1*^CreER/+^*Rosa26*^DTA/+^ mice, with ensuing repopulation deriving from both the proliferation of residual CNS resident microglia and the engraftment of peripheral monocyte-derived microglia-like cells. In this study, tamoxifen was administered to *Cx3cr1*^CreER/+^*Rosa26*^DTA/+^ and *Cx3cr1*^CreER/+^ female and male mice. Experimental autoimmune encephalomyelitis (EAE), a widely used animal model of MS, was induced by active immunization with myelin oligodendrocyte glycoprotein (MOG) one month after tamoxifen injections in *Cx3cr1*^CreER/+^*Rosa26*^DTA/+^ mice and *Cx3cr1*^CreER/+^ mice, a time point when the CNS niche was colonized by microglia derived from both CNS microglia and peripherally-derived macrophages. We demonstrate that engraftment of microglia-like cells following microglial depletion exacerbated EAE in *Cx3cr1*^CreER/+^*Rosa26*^DTA/+^ female mice as assessed by clinical symptoms and the expression of CNS inflammatory factors, but these findings were not evident in male mice. Higher major histocompatibility complex class II expression and cytokine production in the female CNS contributed to the sex-dependent EAE severity in mice following engraftment of microglia-like cells. An underestimated yet marked sex-dependent microglial activation pattern may exist in the injured CNS during EAE.

## 1. Introduction

Multiple sclerosis (MS) is a complex neuroinflammatory disorder of the central nervous system (CNS) associated with progressive and irreversible neurological dysfunctions [1]. As specialized resident innate immune cells in the CNS, microglia provide neurotrophic support, contribute to normal myelinogenesis, synaptic pruning and refine neural circuits, thereby maintaining CNS homeostasis [2,3,4,5]. Microglial activation is widely noted in numerous neurological diseases such as MS [6,7], and comprehensive MS genomic mapping also highlighted the inferred contribution of brain resident microglia [8]. Microglia-induced neuroinflammation plays a fundamental role in both the occurrence and progression of MS [8,9]. It has recently become clear that homeostatic microglia are in fact nearly lost in active and slowly expanding MS lesions, whereas long-lived microglia exhibited an intermediate phenotype between pro-inflammatory and anti-inflammatory states in later stages of MS [10]. The complexity of CNS innate cell populations may be amplified significantly in the context of neuroinflammation, reflecting heterogeneous responses by microglia and subsequent recruitment of diverse circulating immune cells into the injured CNS [11,12]. Considering the complex immunological features of MS, preclinical studies that recapitulate aspects of disease pathogenesis provide a valuable resource for understanding molecular mechanisms and exploring potential therapeutic interventions. Experimental autoimmune encephalomyelitis (EAE), despite several limitations, is the most widely used preclinical research model of MS [13].

How microglia could be precisely targeted for optimal therapeutic efficacy has gained much-deserved attention recently [14,15,16]. Microglia can be depleted experimentally through genetic targeting, conditional genetic targeting or pharmacological therapies as we previously reviewed [17,18]. Several investigations have utilized transgenic microglial depletion animal models such as tamoxifen-induced *Cx3cr1*^CreER^ mice in which diphtheria toxin receptor (DTR) is expressed upon Cre-mediated recombination (*Cx3cr1*^creER^*DTR* mice) [19], and *Cx3cr1*^CreER/+^*Csf1r*^Flox/Flox^ mice that target the CSFR1 receptor, CSFR1 being critical for microglia well-being [20]. Newly repopulated microglia following both genetic and pharmacological depletion exhibit a neuroprotective phenotype and can contribute to recovery after brain injury [21]. More recently, we have demonstrated that microglia can be efficiently depleted by the administration of tamoxifen in *Cx3cr1*^CreER/+^*Rosa26*^DTA/+^ mice, this being followed by simultaneous long-term repopulation of the empty microglial niche from both CNS residual microglia and circulating monocytes [22,23,24]. We and others have proven that circulating monocytes can engraft the CNS and then give rise to long-lived microglia-like cells [20,22]. However, the functionality of cellular therapeutic effects of the engraftment of microglia-like cells during EAE is poorly understood.

We hypothesized that the engraftment of microglia-like cells following experimental microglia depletion could be beneficial for resolving ongoing neuroinflammation and promoting disease recovery in EAE. Immunizing both female and male *Cx3cr1*^CreER/+^*Rosa26*^DTA/+^ mice with myelin oligodendrocyte glycoprotein (MOG) to induce EAE, we report that exacerbated EAE in female mice results from the engraftment of microglia-like cells. This sex-dependent EAE severity following microglia-like cell engraftment may partially be due to higher major histocompatibility complex class II (MHCII) expression and cytokine production in the female CNS.

## 2. Results

### 2.1. Long-Term Engraftment of Microglia-Like Cells in Cx3cr1^CreER/+^Rosa26^DTA/+^ Mice

**Figure 1:**
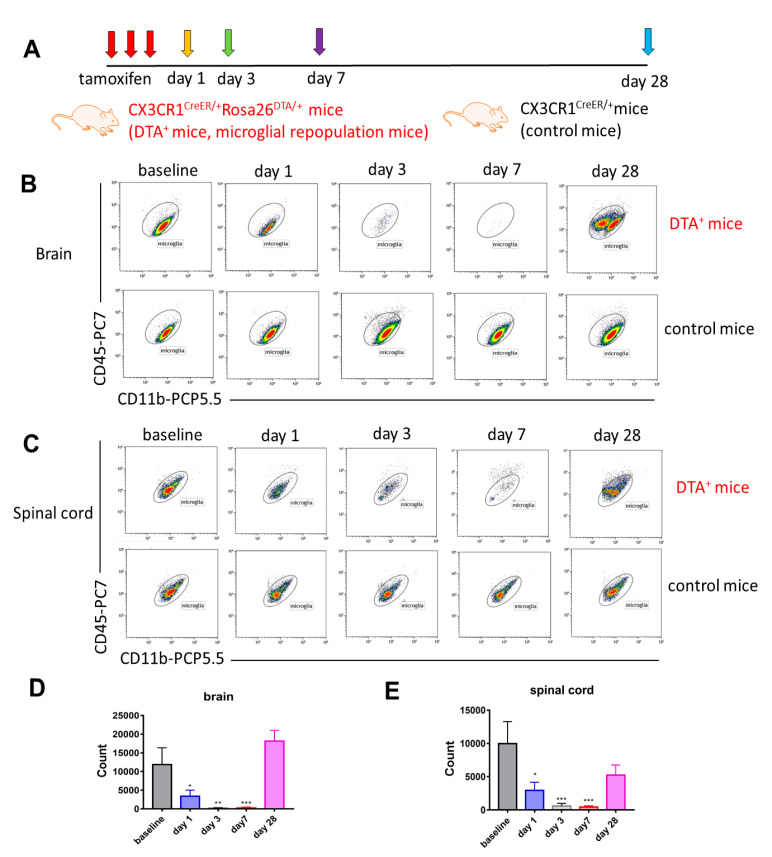
Long-term engraftment of microglia-like cells in *Cx3cr1*^CreER/+^*Rosa26*^DTA/+^ mice (**A**) *Cx3cr1*^CreER/+^*Rosa26*^DTA/+^ and *Cx3cr1*^CreER/+^ mice were sacrificed at different time points (day 1, 3, 7 and 1 month) following three consecutive subcutaneous tamoxifen injections. (**B**,**C**) Representative flow cytometry plots of CD11b^+^CD45^low^Ly6C^−^Ly6G^−^ microglia of the hemi-brains (**B**) and spinal cords (**C**) in *Cx3cr1*^CreER/+^*Rosa26*^DTA/+^ mice (microglial repopulated mice) and *Cx3cr1*^CreER/+^ mice (control mice) are depicted during depletion and repopulation periods. (**D**) Total CD11b^+^CD45^low^Ly6C^−^Ly6G^−^ microglial counts (±SEM) of the hemi-brains and (**E**) spinal cords in *Cx3cr1*^CreER/+^*Rosa26*^DTA/+^ mice during depletion and repopulation periods (baseline, gray bars; day 1, blue bars; day 3, gray bars; day 7, red bars and 1 month, pink bars). *n* = 5, 3, 4, 6 and 3 mice at baseline, day 1, day 3, day 7 and day 28 time points, respectively. Both male and female mice were used. Data are representative of two independent experiments. Statistical significance is indicated as * *p* < 0.05, ** *p* < 0.01 and *** *p* < 0.001.

**Figure 2:**
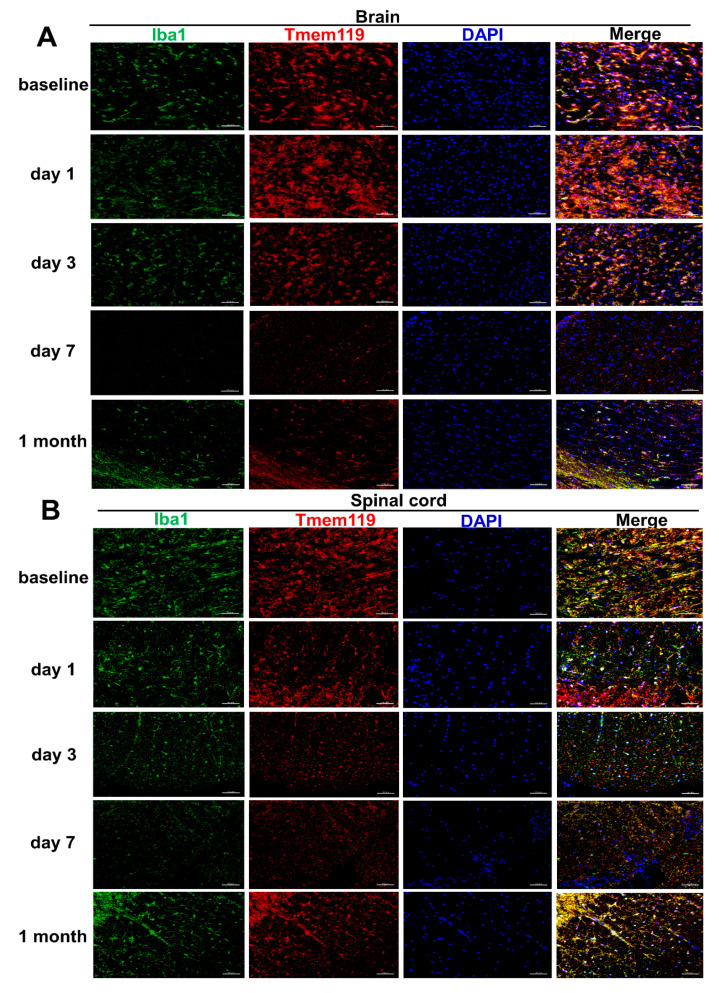
Microglia are effectively depleted by day 7 after tamoxifen injections and repopulated 1 month later in *Cx3cr1*^CreER/+^*Rosa26*^DTA/+^ mice. An example of each time point (baseline, day 1, 3, 7 and 1 month) of double immunofluorescent staining of Iba1 (green) and Tmem119 (red) of *Cx3cr1*^CreER/+^*Rosa26*^DTA/+^ and *Cx3cr1*^CreER/+^ central nervous system (CNS) tissues followed by three consecutive subcutaneous tamoxifen injections. Representative images showed microglia in the cerebral cortex from coronal sections along center line (**A**) and microglia in the spinal cord white matter from mid thoracic transversal sections (**B**). *n* = 5, 3, 4, 6 and 3 mice at baseline, day 1, day 3, day 7 and day 28 time points, respectively. Both male and female mice were used. Data are representative of two independent experiments. Scale bars represent 50 μm.

In order to investigate the kinetic changes of microglial depletion and repopulation *Cx3cr1*^CreER/+^*Rosa26*^DTA/+^ and *Cx3cr1*^CreER/+^ mice were terminated at different time points (day 1, 3, 7 and 1 month) after three consecutive subcutaneous tamoxifen injections (experimental design depicted in Figure 1A), both male and female mice being used. Flow cytometric and immunohistochemical analyses of brain and spinal cord tissues were performed at each time point. Approximately 92% of CD11b^+^CD45^low^Ly6C^−^Ly6G^−^ microglia in the brain (gating strategy presented in Supplementary Figure S1A) could be depleted effectively 7 days after tamoxifen injections. The repopulation of microglia-like cells with two subpopulations, deriving from both the proliferation of residual CNS resident microglia and the engraftment of peripheral monocyte-derived microglia-like cells [21], was evident in the brain one month later (Figure 1B,D). Consistent with the findings in the brain, spinal cord CD11b^+^CD45^low^Ly6C^−^Ly6G^−^ microglia (gating strategy presented in Supplementary Figure S1C) in *Cx3cr1*^CreER/+^*Rosa26*^DTA/+^ mice could also be depleted effectively 7 days after tamoxifen injections, and repopulating microglia-like cells were noted one month later (Figure 1C,E). No depletion of microglia was noted in either the brains or spinal cords of *Cx3cr1*^CreER/+^ mice, indicating their suitability as a control group (Figure 1B,C). We further confirmed these findings by double immunofluorescent staining with Iba1 and Tmem119 antibodies in both brain and spinal cord tissues at different time points (Figure 2A,B). In summary, the engraftment of microglia-like cells occurs in both the brain and spinal cord of *Cx3cr1*^CreER/+^*Rosa26*^DTA/+^ mice one month after administration of tamoxifen.

### 2.2. Female Cx3cr1^CreER/+^Rosa26^DTA/+^ Mice Develop More Severe EAE after Engraftment of Microglia-Like Cells

**Figure 3:**
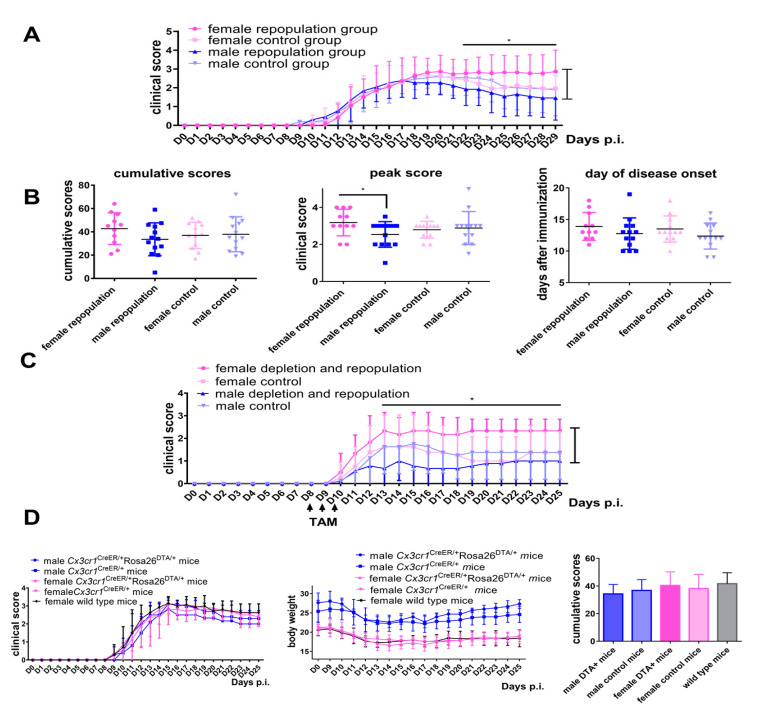
Female *Cx3cr1*^CreER/+^*Rosa26*^DTA/+^ mice develop more severe experimental autoimmune encephalomyelitis (EAE) after engraftment of microglia-like cells (**A**) Tamoxifen was first administered and myelin oligodendrocyte glycoprotein (MOG)-EAE was subsequently induced when microglia were repopulated (one month later). Clinical scores of neurological deficits post-immunization up to 29 days are indicated. Female *Cx3cr1*^CreER/+^*Rosa26*^DTA/+^ mice (*n* = 11) with newly repopulated microglia (dark pink color) had a similar disease onset but experienced a higher disease severity during the chronic stage than did male *Cx3cr1*^CreER/+^*Rosa26*^DTA/+^ mice (*n* = 13) with newly repopulated microglia (dark blue color, * p < 0.05). Female *Cx3cr1*^CreER/+^ mice (*n* = 12) with resident microglia (light pink color) experienced similar clinical scores during the whole EAE period when compared with male *Cx3cr1*^CreER/+^ mice (*n* = 13) with resident microglia (light blue color). Data are representative of two independent experiments. (**B**) Peak disease score, day of disease onset and cumulative scores of both male and female groups are depicted during EAE. (**C**) EAE was first induced and tamoxifen was subsequently administered on days 8, 9 and 10 post-immunization. Clinical scores of neurological deficits post-immunization up to 25 days are presented (*n* = 6, 8, 9, 8, respectively). (**D**) Different mouse strains (*Cx3cr1*^CreER/+^*Rosa26*^DTA/+^ male and female mice, *Cx3cr1*^CreER/+^ male and female mice and *C57BL/6* female wild-type mice, *n* = 3, 9, 5, 5, 7, respectively) without tamoxifen injections developed similar clinical scores and cumulative EAE scores. Statistical significance is indicated as * *p* < 0.05.

EAE was induced by active immunization with MOG one month after tamoxifen injections in *Cx3cr1*^CreER/+^*Rosa26*^DTA/+^ mice and *Cx3cr1*^CreER/+^ mice, a time point when the CNS niche was colonized by microglia derived from both CNS microglia and peripherally-derived macrophages. In female *Cx3cr1*^CreER/+^*Rosa26*^DTA/+^ mice the disease onset was normal but a higher chronic disease severity was recorded than in male mice (Figure 3A,B, * p < 0.05). In contrast, similar clinical scores during the whole EAE observation period were recorded in male and female *Cx3cr1*^CreER/+^ control mice (Figure 3A). Furthermore, days 17–20 correspond to the peak of disease and female *Cx3cr1*^CreER/+^*Rosa26*^DTA/+^ mice had higher peak scores than did male mice (Figure 3B, * p < 0.05), while cumulative scores and day of disease onset were similar between groups. Overall, female *Cx3cr1*^CreER/+^*Rosa26*^DTA/+^ mice developed a more severe EAE course following engraftment of microglia-like cells. In order to further explore if the engraftment of microglia-like cells contributed to disease severity during the EAE recovery period, tamoxifen was administered at days 8–10 post-immunization, allowing microglia-like cells to gradually repopulate after the clinical peak of EAE (Figure 3C). Again, female *Cx3cr1*^CreER/+^*Rosa26*^DTA/+^ mice also experienced a higher severity of the disease than did male mice, while this phenomenon was not evident in *Cx3cr1*^CreER/+^ control mice of either gender (Figure 3C). To exclude that this sex-specific effect of the engraftment of microglia-like cells was not due to a baseline sex difference in EAE development, we also compared the disease courses of male and female mice from the different strains. Our results demonstrate that both males and females, irrespective of their strain background, developed similar EAE courses and cumulative scores (Figure 3D). Taken together, we conclude that engraftment of peripherally-derived microglia-like cells in females exacerbates EAE disease.

### 2.3. CNS Myeloid Cell Compositions during Chronic EAE

**Figure 4:**
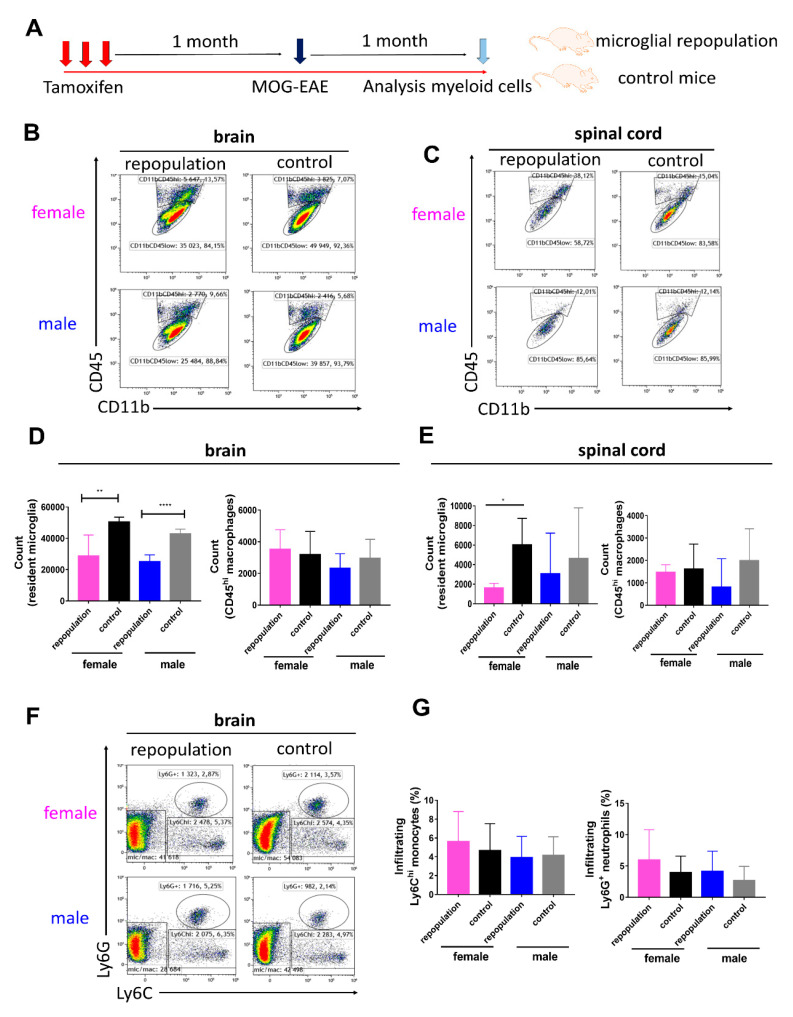
CNS myeloid cell compositions during chronic EAE (**A**) Schematic overview of the experimental design. One month after tamoxifen injections, EAE was induced by active immunization with MOG in *Cx3cr1*^CreER/+^*Rosa26*^DTA/+^ mice with newly repopulated microglia and in *Cx3cr1*^CreER/+^ mice with resident microglia (both male and female mice). Brain and spinal cord tissues were dissected on day 29 post-immunization (chronic EAE period). (**B**,**C**) Myeloid cell subset analysis in the brain (**B**) and spinal cord tissues (**C**) of the *Cx3cr1*^CreER/+^*Rosa26*^DTA/+^ mice and *Cx3cr1*^CreER/+^ mice were performed using flow cytometry. (**D**) The numbers of CD11b^+^CD45^low^Ly6C^−^Ly6G^−^ brain microglia and CD11b^+^CD45^hi^Ly6C^−^Ly6G^−^ macrophages in repopulated and control groups. n  =  5 mice in each group. (**E**) The numbers of CD11b^+^CD45^low^Ly6C^−^Ly6G^−^ spinal cord microglia and CD11b^+^CD45^hi^Ly6C^−^Ly6G^−^ macrophages in repopulated and control groups. *n*  =  3, 5, 4 and 4 mice in female repopulation, female control, male repopulation and male control group, respectively. (**F**) Representative flow cytometry plots of infiltrating Ly6C^+^ monocytes and Ly6G^+^ neutrophils in EAE brain tissues are depicted during the chronic EAE period. (**G**) Infiltrating Ly6C^+^ monocyte and Ly6G^+^ neutrophil proportions in EAE brains did not differ among different groups during chronic EAE period. Statistical significance is indicated as * *p* < 0.05, ** *p* < 0.01 and *** *p* < 0.001.

We further confirmed that microglia depletion followed similar dynamics between male and female *Cx3cr1*^CreER/+^*Rosa26*^DTA/+^ mice (Supplementary Figure S2A) and the sex differences in EAE severity were not dependent on the overall number of repopulating microglia in *Cx3cr1*^CreER/+^*Rosa26*^DTA/+^ mice (Supplementary Figure S2B). In order to analyze sex differences of CNS myeloid cell compositions during the chronic EAE stage, brain and spinal cord tissues were dissected 1 month post-immunization (schematic overview is depicted in Figure 4A). Myeloid cell compositions in the brain and spinal cord tissues of *Cx3cr1*^CreER/+^*Rosa26*^DTA/+^ mice and *Cx3cr1*^CreER/+^ mice were analyzed using flow cytometry. The gating strategies of brain and spinal cord myeloid cells during EAE are depicted in Supplementary Figure S1B,D, respectively. We observed that infiltrating CD11b^+^CD45^hi^Ly6C^−^Ly6G^−^ macrophages, Ly6C^+^ monocytes and Ly6G^+^ neutrophils were still present in the CNS tissues during the chronic EAE stage (Figure 4B,C,F). Newly repopulated CD11b^+^CD45^low^Ly6C^−^Ly6G^−^ microglia in the brains were lower in number than in control groups (Figure 4D). A similar trend was also recorded in the spinal cord (Figure 4E). Infiltrating CD11b^+^CD45^hi^Ly6C^−^Ly6G^−^ macrophages in the CNS tissues did not differ among different groups (Figure 4D,E). The percentage of infiltrating Ly6C^+^ monocytes and Ly6G^+^ neutrophils in chronic EAE brains and spinal cords did not differ between *Cx3cr1*^CreER/+^*Rosa26*^DTA/+^ and *Cx3cr1*^CreER/+^ mice or between sexes (Figure 4F,G).

### 2.4. Higher MHC II Expression of Infiltrating Ly6C^hi^ Monocytes during Peak EAE in Female Microglia-Repopulated Mice

**Figure 5:**
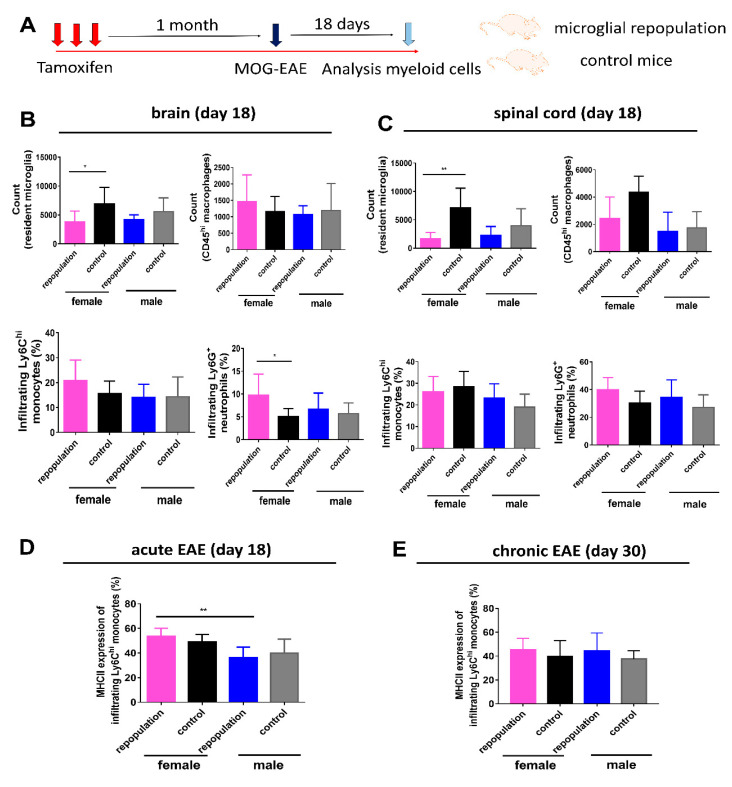
Higher MHC II expression of infiltrating Ly6C^hi^ monocytes during peak EAE in female microglia-repopulated mice (**A**) Schematic overview of the experimental design. One month after tamoxifen injections, EAE was induced by active immunization with MOG in *Cx3cr1*^CreER/+^*Rosa26*^DTA/+^ mice with newly repopulated microglia and *Cx3cr1*^CreER/+^ mice with resident microglia (both male and female mice). Brain and spinal cord tissues were dissected on day 18 post-immunization (acute EAE period). Myeloid cell subset analysis in the brain (**B**) and spinal cord tissues (**C**) of *Cx3cr1*^CreER/+^*Rosa26*^DTA/+^ mice and *Cx3cr1*^CreER/+^ mice were performed using flow cytometry during the acute EAE disease course. MHC II expression (%) of infiltrating Ly6C^hi^ monocytes was higher in the female repopulated microglia group than in the male repopulated group during the acute EAE period (**D**), while significant difference was not evident during the chronic EAE period (**E**). Acute EAE experiment: *n*  =  6 mice/group in female repopulation, female control and male repopulation groups, and *n*  =  7 mice in male control group. Chronic EAE experiment: *n*  =  5 mice in each group. Statistical significance is indicated as * *p* < 0.05 and ** *p* < 0.01.

We next explored CNS myeloid cells during the acute EAE stage. Brain and spinal cord tissues were thus dissected on day 18 post-immunization (schematic overview is depicted in Figure 5A) and sex differences were also noted in this setting (Supplementary Figure S3). Consistent with previous EAE results, there was a trend that following engraftment of microglia-like cells female *Cx3cr1*^CreER/+^*Rosa26*^DTA/+^ mice developed worse clinical symptoms than in *Cx3cr1*^CreER/+^ mice with resident microglia (Supplementary Figure S3A). *Cx3cr1*^CreER/+^*Rosa26*^DTA/+^ female mice had an earlier disease onset than that of female control mice, and both peak score and cumulative scores were significantly higher than in female control mice (Supplementary Figure S3B), while the corresponding male groups were comparable (Supplementary Figure S3B). Furthermore, CD11b^+^CD45^low^Ly6C^−^Ly6G^−^ microglia in both the brain and spinal cord were less numerous in microglia-like cell engrafted groups than in control groups (Figure 5B,C). Infiltrating CD11b^+^CD45^hi^Ly6C^−^Ly6G^−^ macrophages in the brain EAE tissues did not differ among groups (Figure 5B,C), while infiltrating Ly6G^+^ neutrophils in EAE brains was significantly increased in the female repopulated microglia group than in the female control group (Figure 5B). Proportions of infiltrating Ly6C^+^ monocytes and Ly6G^+^ neutrophils in EAE spinal cord tissues did not differ among groups during the acute EAE period (Figure 5C). Importantly, MHC II expression of infiltrating Ly6C^hi^ monocytes was significantly increased in female repopulated microglia mice than in the male group during the acute EAE period, but not during the chronic EAE period (Figure 5D,E).

### 2.5. Elevated Cytokine Production during EAE Peak in Female Microglia-Repopulated Mice

**Figure 6:**
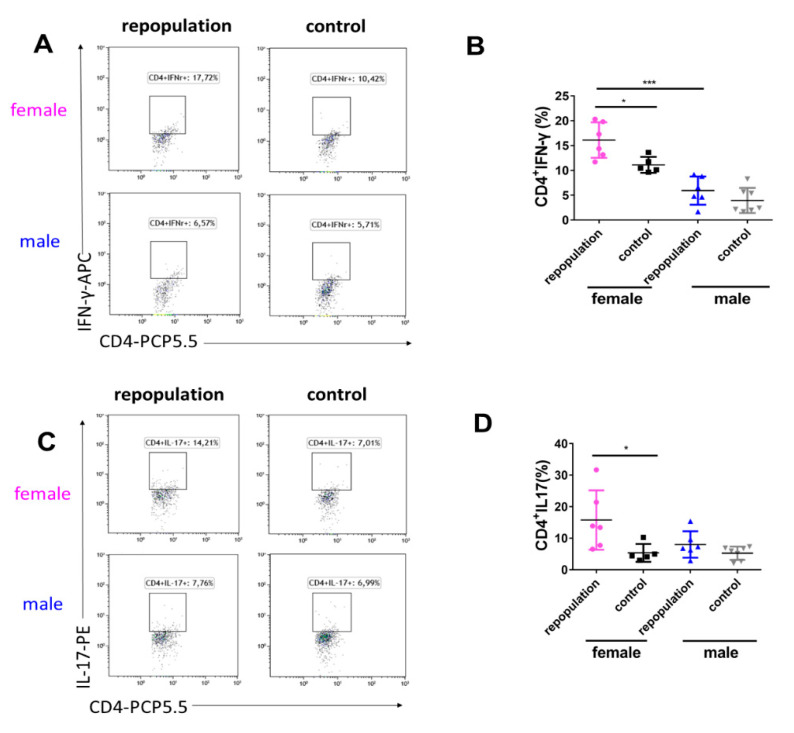
Elevated cytokine production during EAE peak in female microglia-repopulated mice. (**A**,**B**) Flow cytometry data showing the expression of IFN-γ in CD4^+^ T cells from the brains of *Cx3cr1*^CreER/+^*Rosa26*^DTA/+^ and *Cx3cr1*^CreER/+^ mice. (**C**,**D**) Flow cytometry data showing the expression of IL-17 in CD4^+^ T cells from the brains of *Cx3cr1*^CreER/+^*Rosa26*^DTA/+^ and *Cx3cr1*^CreER/+^ mice. (B,D) Representative numbers indicate the percentage of each compartment. *n*  =  6, 5, 6 and 7 mice in female repopulation, female control, male repopulation and male control groups, respectively. Statistical significance is indicated as * *p* < 0.05 and *** *p* < 0.001.

In concert with the higher clinical score, we observed that the expression of Interferon-γ (IFN-γ) in CD4^+^ T cells (CD4^+^IFN-γ^+^ T cell subgroup) was significantly greater in female *Cx3cr1*^CreER/+^*Rosa26*^DTA/+^ brains than in both the female control group and male *Cx3cr1*^CreER/+^*Rosa26*^DTA/+^ brains during the acute EAE stage (Figure 6A,B). Furthermore, the expression of IL-17 in CD4^+^ T cells (CD4^+^IL-17^+^ T cell subgroup) was also significantly higher in female *Cx3cr1*^CreER/+^*Rosa26*^DTA/+^ brains when compared to female control mice. (Figure 6C,D). Furthermore, no sex differences in proportions and proliferation of infiltrating CD4^+^ and CD8^+^ T cells in the brain (18 days post-immunization of EAE) were noted in *Cx3cr1*^CreER/+^*Rosa26*^DTA/+^ mice and *Cx3cr1*^CreER/+^ mice (Supplementary Figure S4). These data suggest that higher cytokine production in the female brains with microglia-like cells contributed to the sex-dependent exacerbation of EAE.

## 3. Discussion

In this study we utilized the *Cx3cr1*^CreER/+^*Rosa26*^DTA/+^ mouse model which has the advantage of no need of invasive administration of diphtheria toxin, as is the case in the *Cx3cr1*^CreER^:iDTR animal model [19]. Our previous studies using this model demonstrated that newly repopulating microglia are a combination of repopulation from both self-renewing microglia and of infiltrating monocytes that undergo transcriptional modification to become microglia-like cells [22,24]. In the present study we addressed the functionality of these engrafted microglia-like cells in the setting of neuroinflammatory EAE disease, noting their reduced ability to limit pathology compared to self-renewing microglia. The functional loss of microglial homeostasis or microglial trophic factors is thus not beneficial for EAE disease recovery. The significantly increased injury was sex-dependent, with females experiencing worse clinical symptoms. This suggests that there are sex-specific differences in microglia-like cells. Recent data obtained from additional preclinical models also indicated that the number and phenotype of microglia may differ between females and males [25,26]. Microglial functional differences may predispose to previously underestimated but marked sex-dependent microglial activation patterns and signaling cascades in settings of CNS damage [27]. However, there is an argument that MOG-EAE is considered less valid as an animal model for MS since MOG-antibody-associated disease is now considered as a new antigen-specific neuroimmunological disorder [28]. It should be noted that the complex pathological MS processes depend on a combination of the genetic diversity of individuals, a wide range of environmental factors and different immune cell types in humans, while its animal model EAE may not cover the entire spectrum of these characteristics. As the implementation of microglial depletion and repopulation is currently not initiated in patients with MS or MOG-antibody-associated disease, the EAE model provides an opportunity to address the concept. Our study extends the understanding of the distinct functions of engrafted male and female microglia-like cells following depletion, and might provide valuable insights for the decision-making for female vs. male MS patients when microglial-depletion therapies eventually become available.

The timing of microglial ontogeny and development places them in a special position compared with other tissue resident macrophages. Microglia can be self-maintained by local proliferation and apoptosis with little contribution from peripherally circulating monocytes in physiological conditions. We and others have previously demonstrated that peripherally-derived macrophages can give rise to engrafted long-lived microglia-like cells in the brain parenchyma using experimental models, with this new phenotype retaining distinct gene signatures and functional differences such as phagocytic capacity and responses to in vitro challenges [20,22,29]. The functional differences between these engrafted microglia-like cells and yolk sac-derived resident microglia in vivo thus have clinical implications for the treatment of diverse neurological diseases, as we have previously reviewed [30]. It is also important to note that CX3CR1^+^ resident macrophages such as perivascular, subdural meningeal and choroid plexus macrophages within the CNS might also be affected in *Cx3cr1*^CreER/+^*Rosa26*^DTA/+^ mice after treating with tamoxifen [31,32]. These cell populations play a complex role in the pathogenesis of EAE, but it is difficult to distinguish them from infiltrating macrophages using surface markers. Nevertheless, by using our gating strategy we referred to the CD11b^high^CD45^high^ population as ‘infiltrating macrophages’ since in the context of EAE, the contribution of infiltrating macrophages is more dominant compared to that of the resident CNS macrophage pool; CNS-resident macrophages expand modestly compared to the considerable infiltration of peripheral monocytes/macrophages. Although the CNS tissues were perfused thoroughly with PBS and dissected carefully from the meninges for flow cytometrical analysis to minimize the potential contribution from CNS-resident macrophages, the results still need to be interpreted with caution. Technically, applying advanced methods including single-cell RNA-sequencing, high-dimensional cytometry and fate-mapping or using transgenic tools such as *Cx3cr1*^CreERT2^*R26td*^Tomato^ mice to label resident macrophages may provide valuable information regarding the relative contribution of these border-associated macrophages in the context of EAE between male and female.

Recent data indicated that microglial repopulation following experimental depletion using pharmacological colony-stimulating factor 1 receptor (CSF1R) inhibition could effectively resolve inflammation and promote disease recovery after brain injury, without any apparent adverse effects [33]. Using the same approach, age-related spatial memory impairment, microglial cell densities and morphologies could all be reversed by replacing primed microglia in aged mice with newly repopulated microglia [34], and they are also crucial for CNS regeneration following pro-inflammatory microglial necroptosis in a focal demyelinated animal model [35]. Furthermore, bone marrow-derived microglia-like cells may be more efficient in clearing amyloid beta deposits than their CNS endogenous counterparts [36]. CSF1R inhibition-mediated repopulation largely promotes repopulation by self-renewing microglia, while engraftment of microglia-like cells occurs in our *Cx3cr1*^CreER/+^*Rosa26*^DTA/+^ mice. However, we did not observe a neuroprotective effect followed by microglial depletion and repopulation in our case. Different experimental depletion methods might explain some of the discrepancies. Long-term systematic administration of CSF1R inhibitors may affect other immune cells expressing CSF1R in the periphery, then generally suppressing immune responses [18]. In *Cx3cr1*^CreER/+^*Rosa26*^DTA/+^ mice, tamoxifen injections may thus modulate systemic immune responses during EAE course (Figure 3C). Furthermore, myeloid cells may also play regulatory roles in EAE and in our model, the overall number of microglia was fewer than the control group one month after experimental depletion. The relative importance of microglial subpopulations such as resident microglia, disease-associated microglia and peripheral-engrafted microglia-like cells during microglial depletion and repopulation periods at different disease stages needs to be explored and validated using different depletion methods in the future.

Tamoxifen, a non-steroidal estrogen receptor modulator, is used as a hormone therapy drug for treating breast cancer. Previous studies have reported that low doses of tamoxifen may promote microglial polarization toward an anti-inflammatory phenotype in male mice following chronic hypoperfusion [37]. Some may argue that tamoxifen itself may exert confounding effects on EAE independent of microglia depletion and repopulation. We induced MOG-EAE almost one month after injecting tamoxifen, and this should minimize any potential impact of tamoxifen on disease development. In our study we also included male and female EAE mice that were not treated with tamoxifen, and these developed comparable clinical scores during EAE, suggesting no sex difference in wild-type mice. Taken together, our data support the notion that the replacement of microglia by engraftment of microglia-like cells has a sex-specific consequence on development of autoimmune neuroinflammation.

Our results revealed that no sex differences in proportions and proliferation of infiltrating CD4^+^ and CD8^+^ T cells in the brain (18 days post-immunization of EAE) were evident between *Cx3cr1*^CreER/+^*Rosa26*^DTA/+^ mice and *Cx3cr1*^CreER/+^ mice. It is now well accepted that activated microglia and infiltrating T cells interact during disease conditions, and our results suggested that the sex differences in EAE severity might not depend on the changes of CD4^+^ T cells or CD8^+^ T cells after the engraftment of microglia-like cells. Antigen can be presented by microglia via MHC II. Resident microglia and peripheral-derived microglia-like cells may perform specialized distinct functions in vivo. In our study the gender-dependent EAE severity in *Cx3cr1*^CreER/+^*Rosa26*^DTA/+^ mice with engraftment of microglia-like cells may be partially due to their higher MHC II expression and cytokine production in the female CNS. Correlation with enhanced CD4^+^IFN-γ^+^ and CD4^+^IL-17^+^ functionality in female mice supports this increased disease-promoting immune activity.

Our study has several limitations, such as low yield of cells from spinal cords following enzymatic digestion for intracellular/cytokines staining. It also hampers the future investigation of the potential differences in cellular phenotypes and biological functions of repopulated microglia between brain and spinal cord, as suggested by other researchers [38]. Further investigating inflamed spinal cords in EAE may provide additional cues of the sex-specific effects of peripheral microglia-like cell engraftment during neuroinflammation. Although we still noted the sex-specific effects during acute EAE, a larger sample size will be needed to validate our findings in the future. With the engrafted microglia-like cells inflammatory mediators may be changed differentially between males and females during the acute period, then contributing to an obvious sex-specific effect during the disease recovery period.

There is a current notion that peripherally-derived macrophages could be used to replace defective microglia and thus may have therapeutic potential for a range of CNS pathologies [34,36]. Our study indicates that careful consideration of gender effects and of desired in vivo functionality should be taken in design of future microglial replacement therapies.

## 4. Materials and Methods

### 4.1. Ethics Statement

All experiments in this study were approved and performed in accordance with the guidelines from the Swedish National Board for Laboratory Animals and the European Community Council Directive (86/609/EEC) and the local ethics committee of Stockholm North under the ethical permits 9328-2019. All efforts were made to minimize animal suffering and discomfort.

### 4.2. Animals

*Cx3cr1*^CreER^ (Jax Stock: 021160) and *Rosa26*^DTA^ (Jax Stock: 010527) mice were purchased from the Jackson Laboratory and bred to obtain both *Cx3cr1*^CreER/+^*Rosa26*^DTA/+^ and *Cx3cr1*^CreER/+^ mice that were used for experiments. *C57BL/6NTac* mice (Taconic) were bred in the Comparative Medicine Department at Karolinska University Hospital, Sweden. All experimental mice were maintained under a regulated light/dark schedule and temperature conditions of this specific pathogen-free animal facility. All experimental mice were aged between 5 and 13 weeks-old and had free access to standard rodent chow and water.

### 4.3. Tamoxifen Treatment

In order to induce the Cre recombinase in *Cx3cr1*^CreER/+^*Rosa26*^DTA/+^ mice, treatment with tamoxifen (TAM; Sigma, T5648-1G, St Louis, USA) was conducted when mice were 5–7 weeks old. Tamoxifen was resuspended in corn oil (Sigma, C8267-500ML, St Louis, MO, USA) at 75 °C for at least 60 min. The *Cx3cr1*^CreER/+^*Rosa26*^DTA/+^ and *Cx3cr1*^CreER/+^ mice were administered 5 mg (200 μL) TAM subcutaneously on three consecutive days for microglial depletion, and then kept for one month to permit microglial repopulation, as we previously described [21,22].

### 4.4. EAE Induction and Clinical Evaluation

EAE was induced based on the standard protocol in our lab [39]. Briefly, MOG (amino acids 1–125 from the N terminus) was expressed in *Escherichia coli* and purified to homogeneity by chelate chromatography. Purified MOG dissolved in 6M urea was then dialyzed against sodium acetate buffer (10 mM, pH 3.0) to obtain a soluble preparation. Mice were anesthetized with isoflurane (Forane; Abbott Laboratories, Abbot Park, IL, USA) and injected subcutaneously in the dorsal tail base with 35 μg of MOG in phosphate-buffered saline emulsified in Complete Freund’s Adjuvant (CFA, Chondrex, Inc, 7027, Redmond, WA, USA) containing 100 μg heat-killed Mycobacterium tuberculosis H37Ra (Difco Laboratory, Detroit, MI, USA). On the day of immunization and 48h later, mice were injected intraperitoneally with 200 ng pertussis toxin (Sigma, P7208-50UG, St Louis, MO, USA). Body weight and paralytic symptoms were assessed daily from 8 days post-immunization. The clinical signs of EAE were scored according to the following criteria: 0, no clinical signs of EAE; 1, tail weakness or tail paralysis; 2, hindlimb paraparesis or hemiparesis; 3, hindlimb paralysis or hemiparalysis; 4, tetraplegia or moribund; and 5, death (0.5 being assigned for intermediate clinical signs). The cumulative score was calculated as the sum of the daily scores of individual mouse from day 0 until the day of termination. The clinical symptoms were scored in a blinded manner by 3 independent researchers.

### 4.5. Preparation of Single Cell Suspensions from CNS Tissues

Mice were deeply anaesthetized by injecting 100 μg pentobarbital intraperitoneally. Mice were perfused through the left cardiac ventricle using ice-cold PBS. Whole brain and spinal cord were removed and minced using a surgical disposable scalpel (MediCarrier AB, Stockholm, Sweden), followed by enzymatic digestion using Collagenase (11088866001, Roche, Sweden) and DNAse (000000010104159001, Roche, Sweden). Myelin was removed using a 38% Percoll gradient (P1644-1L, Sigma, Sweden).

### 4.6. Flow Cytometry

Single cell suspensions were plated into 96-well V-bottom plates and stained at 4 °C for 20 min. Dead cells were removed using Live/Dead Fixable Near-IR Dead Cell Stain Kit (Invitrogen, Thermo Fisher Scientific, Stockholm, Sweden) in each panel. The following antibody panels were used: 1. For myeloid cells analysis, single cell suspensions were incubated with Percp-Cy5.5-CD11b (clone: M1/70, BioLegend, San Diego, USA), PE/Cy7-CD45 (clone: 30-F11, BioLegend, San Diego, CA, USA), PE-Ly6C (clone: HK1.4, BioLegend, San Diego, CA, USA), V450-Ly6G (clone: 1A8, BD Biosciences, Sweden), APC-F4/80 (clone: BM8, BioLegend, San Diego, CA, USA), Alexa Fluor700-MHCII (clone: M5/114.15.2, BioLegend, San Diego, CA, USA). 2. For T cells analysis, single cell suspensions were incubated with FITC-CD4 (clone: RM4-5, BD Biosciences, Sotckholm, Sweden), PE-CD8 (clone: 53-6.7, eBioscience, San Diego, CA, USA), Percp-Cy5.5-CD3 (clone: 145-2C11, BioLegend, San Diego, CA, USA), APC-CD62L (clone: MEL-14, eBioscience, San Diego, CA, USA), Alexa Fluor700-CD44 (clone: IM7, BioLegend, San Diego, CA, USA), PE/Cy7-Foxp3 (clone: FJK-16s, eBioscience, San Diego, CA, USA) and V450-Ki67 (clone: B56, BD Biosciences, Stockholm, Sweden). 3. In order to measure intracellular cytokines, brain cells were seeded into 96-well plates with 200 µl of complete Dulbecco’s Modified Eagle Medium (DMEM) containing 10% fetal bovine serum (BCCB2249, Sigma, Malmö, Sweden), penicillin-streptomycin (048M4774V, Sigma, Sweden), L-glutamine (G7513, Sigma, Malmö, Sweden), 2-mercaptoethanol (Gibco by Life Technologies, Stockholm, Sweden) and sodium pyruvate (11360039, Thermo Fisher Scientific, Stockholm, Sweden) and stimulated with Ionomycin (I0634-1MG, Sigma-Aldrich, Malmö, Sweden), PMA (P1585-1MG, Sigma-Aldrich, Malmö, Sweden) and GolgiPlug (555029, BD Biosciences, Stockholm, Sweden) for 5–6 h. Afterwards, brain cells were first incubated with Alexa PCP5.5-CD4 (clone: GK15, BioLegend, San Diego, CA, USA), PE/Cy7-CD8 (clone: 53-6.7, BioLegend, San Diego, CA, USA) and A700-CD3 (clone: 17A2, BioLegend, San Diego, CA, USA). Then cells were treated with fixation/permeabilization buffer (eBioscience, San Diego, CA, USA) at least 30 min and followed by intracellular staining Fluor488-IL-4 (clone: 11B11, BioLegend, San Diego, CA, USA), PE-IL-17 (clone: TC11-18H10, BD Biosciences, Stockholm, Sweden) and APC-IFN-γ (clone: XMG1.2, BD Biosciences, Stockholm, Sweden). Cells were acquired using a Gallios flow cytometer (Beckman Coulter) and analyzed using Kaluza software (Beckman Coulter).

### 4.7. CNS Histology and Immunohistochemistry

Brain and spinal cord tissues were dissected after perfusion using ice-cold PBS, and then immersed in 4% paraformaldehyde (PFA, Histolab, Gothenburg, Sweden) overnight, followed by cryoprotection with 30% sucrose in 0.1 M phosphate buffer at 4 °C. Immunohistochemistry of the hemi-brain tissues was conducted in free-floating sections. Brain tissues were sectioned onto 25 μm slides using a Leica SM 2010 R Sliding Microtome (or a Leica SM 2000 R Sliding Microtome, Leica, Wetzlar, Germany) and stored in tissue cryoprotectant solution (25% ethylene glycol and 25% glycerin in 0.1M phosphate buffer). Sections were washed in Tris-buffered saline (TBS, 50mM Tris-HCl in 150mM NaCl, pH 7.5) and then blocked with 3% donkey serum in TBS with 0.1% Triton X-100 under moderate shaking. After blocking, brain sections were incubated overnight with the following primary antibodies: Goat anti-Iba-1 (1:500, ab5076, Abcam, Cambridge, MA, USA) and Rabbit anti-Tmem119 (1:500, ab209064, Abcam, Cambridge, MA, USA). Thereafter, sections were rinsed with TBS and incubated for 2 h at room temperature with the indicated second antibodies: Donkey anti Goat (1:1000, CF®633, #20127, BIOTIUM, Fremont, CA, USA) and Donkey anti-rabbit (1:1000, Alexa Fluor 555, Thermo Fisher Scientific, Stockholm, Sweden) together with Hoechst (1:1000, #33342, Thermo Fisher Scientific, Stockholm, Sweden). Sections were mounted and coverslipped using ProLong^®^ Gold anti-fade reagent (P10144, Thermo Fisher Scientific, Stockholm, Sweden). Spinal cord tissues sections (25 μm thick) were prepared using a Leica CM3050 S Research Cryostat (or Leica CM1850 Research Cryostat, Leica, Wetzlar, Germany) and mounted onto Superfrost^®^ glass slides (J1800AMNT, Thermo Fisher Scientific, Stockholm, Sweden). Fluorescent images were captured using a LSM700 laser scanning confocal microscope (Axio-observer Z1; CarlZeiss microscopy, Wetzlar, Germany), and analyzed using ZEN software (the black edition; Zeiss).

### 4.8. Statistical Analysis

Statistical analysis was conducted using GraphPad software 8 (San Diego, CA, USA). After examining the normal distribution, the clinical scores were analyzed using a two-way ANOVA followed by Tukey’s post hoc test. Data for multiple variable comparisons were analyzed using the Kruskal–Wallis test. Comparisons between two groups were made with Mann–Whitney tests. Error bars are presented as SEM. Differences at *p* < 0.05 were considered to be statistically significant.

## 5. Conclusions

The engraftment of microglia-like cells following microglial depletion exacerbated EAE in females. An underestimated yet marked sex-dependent microglial activation pattern may exist in the injured CNS during EAE.

## Supplementary Materials

Supplementary Materials can be found at https://www.mdpi.com/1422-0067/21/18/6824/s1.

## Abbreviations

CNS	central nervous system
CSF1R	colony-stimulating factor 1 receptor
DTR	diphtheria toxin receptor
EAE	experimental autoimmune encephalomyelitis
IFN-γ	interferon-γ
MHCII	major histocompatibility complex class II
MOG	myelin oligodendrocyte glycoprotein
MS	multiple sclerosis
