# Supplementary material for: Sex-Specific Effects of Microglia-Like Cell Engraftment during Experimental Autoimmune Encephalomyelitis
Source: Int J Mol Sci. 2020 Sep 17;21(18):6824. doi: 10.3390/ijms21186824 (PMC7555782; doi:10.3390/ijms21186824)
Supplement: Supplementary file 1 [file ijms-21-06824-s001.pdf]

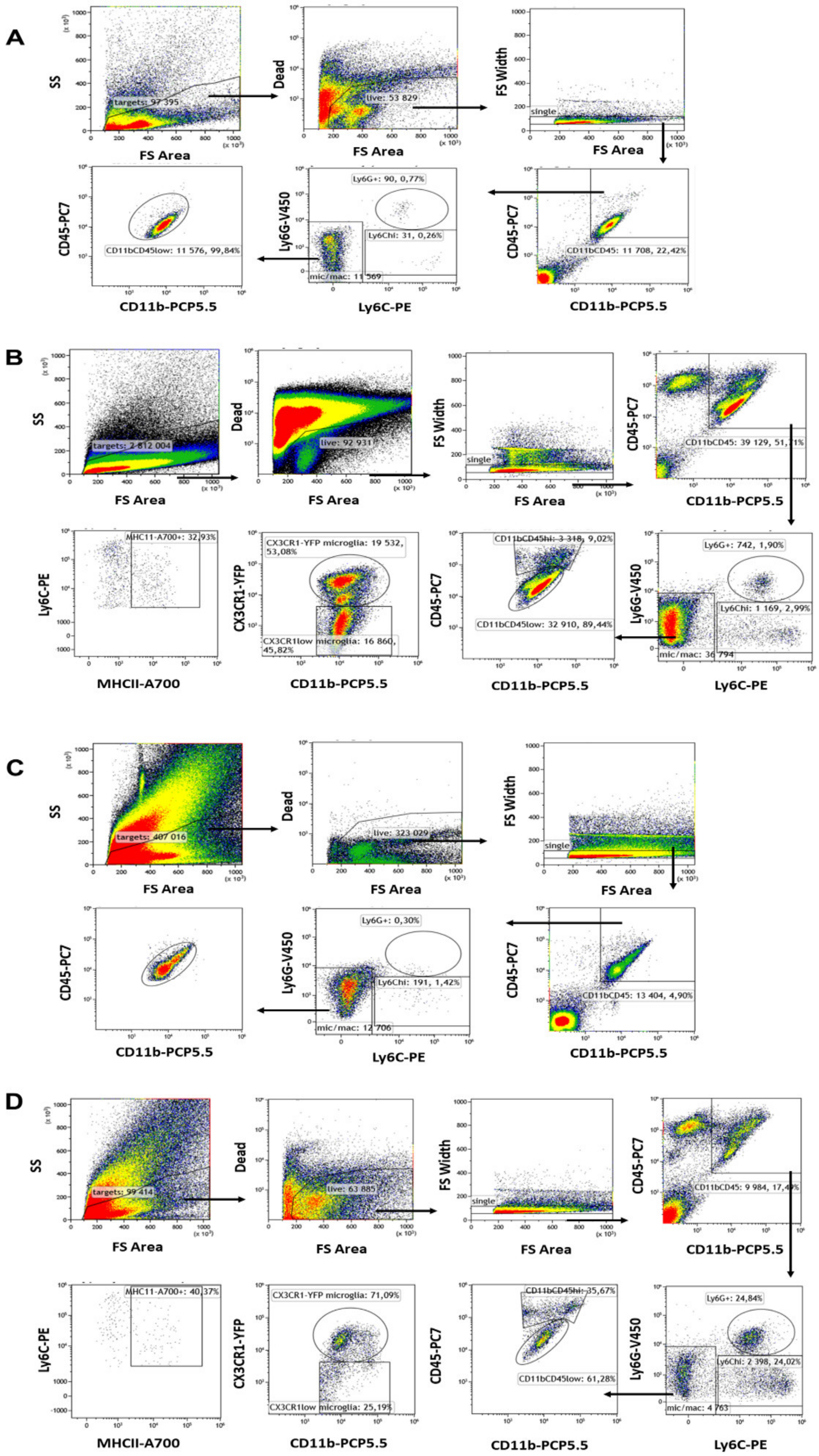
A
SS
FS Area
targets: 97 395
Dead
live: 53 829
FS Width
single
CD45-PC7
CD11bCD45: 11 708, 22,42%
CD11b-PCP5.5
Ly6G-V450
Ly6G+: 90, 0,77%
Ly6Chi: 31, 0,26%
mic/mac: 11 569
Ly6C-PE
CD11bCD45low: 11 576, 99,84%
B
targets: 2 812 004
live: 92 931
CD11bCD45: 39 129, 51,71%
Ly6G+: 742, 1,90%
Ly6Chi: 1 169, 2,99%
mic/mac: 36 794
CD11bCD45hi: 3 318, 9,02%
CD11bCD45low: 32 910, 89,44%
CX3CR1-YFP
CX3CR1-YFP microglia: 19 532, 53,08%
CX3CR1low microglia: 16 860, 45,82%
MHC11-A700+: 32,93%
MHCII-A700
C
targets: 407 016
live: 323 029
CD11bCD45: 13 404, 4,90%
Ly6G+: 0,30%
Ly6Chi: 191, 1,42%
mic/mac: 12 706
D
targets: 99 414
live: 63 885
CD11bCD45: 9 984, 17,49%
Ly6G+: 24,84%
Ly6Chi: 2 398, 24,02%
mic/mac: 4 763
CD11bCD45hi: 35,67%
CD11bCD45low: 61,28%
CX3CR1-YFP microglia: 71,09%
CX3CR1low microglia: 25,19%
MHC11-A700+: 40,37%

Supplementary Figure 1: Gating strategy for flow cytometric analysis (A) Gating strategy of brain myeloid cells in normal *Cx3cr1*$^{CreER/+}$*Rosa26*$^{DTA+}$ mice without microglial depletion and repopulation. **(B)** Gating strategy of brain myeloid cells during MOG-induced EAE in *Cx3cr1*$^{CreER/+}$*Rosa26*$^{DTA+}$ mice with newly repopulated microglia. **(C)** Gating strategy of spinal cord myeloid cells in *Cx3cr1*$^{CreER/+}$*Rosa26*$^{DTA+}$ mice without microglial depletion and repopulation. **(D)** Gating strategy of spinal cord myeloid cells in MOG-induced EAE *Cx3cr1*$^{CreER/+}$*Rosa26*$^{DTA+}$ mice with newly repopulated microglia.

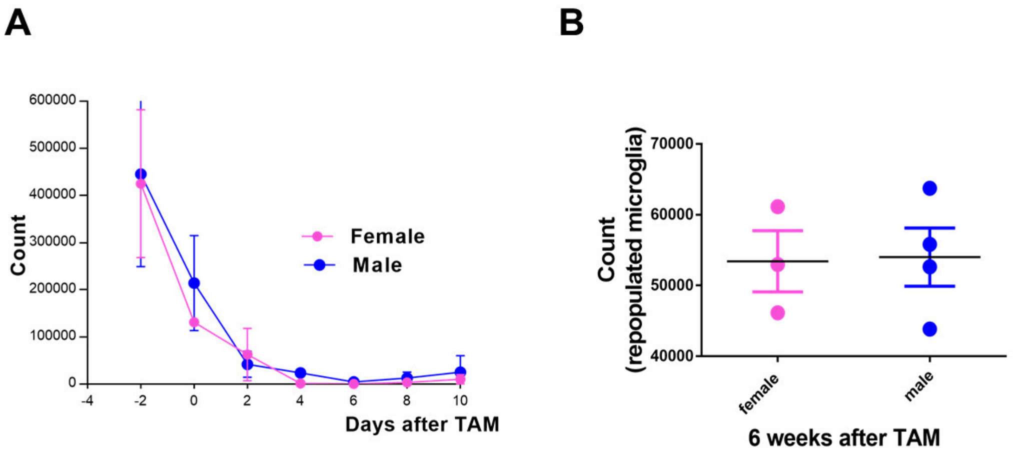

Supplementary Figure 2: (A) Microglial depletion follows similar dynamics between male and female *Cx3cr1*$^{CreER/+}$*Rosa26*$^{DTA/+}$ mice at relevant time points. (B) There are no differences regarding the overall numbers of repopulating microglia 6 weeks after tamoxifen injections.

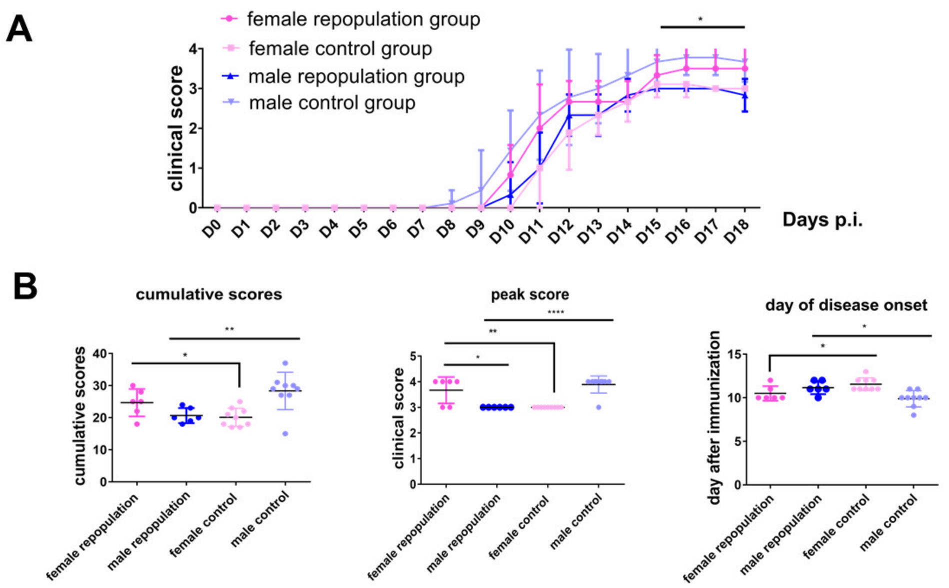

Supplementary Figure 3: Sex-specific effects of microglia-like cell engraftment during the acute EAE phase. (A) Clinical scores of neurological deficits post-immunization up to 18 days are indicated. (B) Cumulative scores, peak disease score and day of disease onset in both male and female groups are depicted.

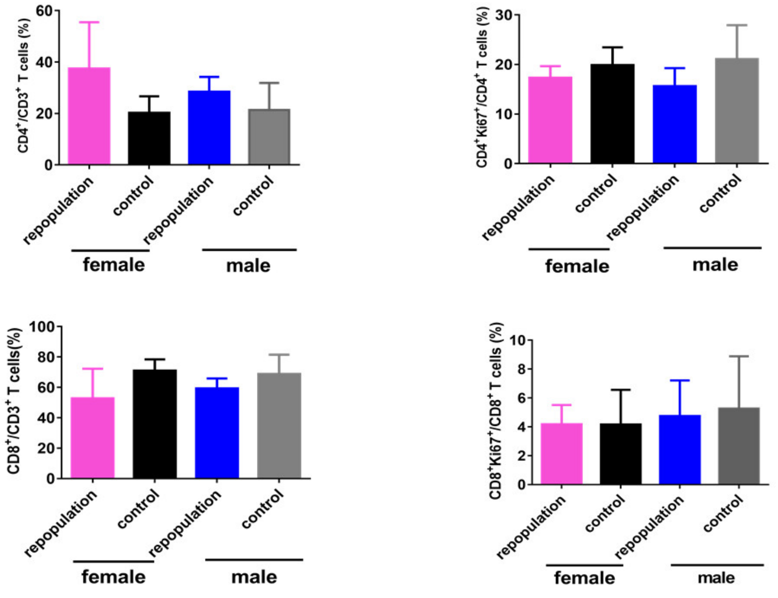

Supplementary Figure 4: No differences of infiltrating T cells between sexes during acute EAE (18 days post-immunization of EAE) were recorded in *Cx3cr1*$^{CreER/+}$*Rosa26*$^{DTA/+}$ mice with repopulated microglia and *Cx3cr1*$^{CreER/+}$mice with resident microglia. $n = 6$ mice/group in female repopulation, female control and male repopulation groups, and $n = 7$ mice in male control group.
